# Occurrence mechanism and coping paths of accidents of highly aggregated tourist crowds based on system dynamics

**DOI:** 10.1371/journal.pone.0222389

**Published:** 2019-09-17

**Authors:** Jie Yin, Xiang-min Zheng, Ruey-Chyn Tsaur

**Affiliations:** 1 College of tourism, Huaqiao University, Quanzhou, Fujian Province, China; 2 Department of Management Sciences, Tamkang University, New Taipei City, Taiwan; Fukushima Medical University School of Medicine, JAPAN

## Abstract

The safety of highly aggregated tourist crowds is a challenging and important issue. This paper not only provided a comprehensive analysis of the accidents of highly aggregated tourist crowds but also determined the occurrence mechanism and coping paths. Based on the analysis of multiple cases, we found that the variable status of highly aggregated tourist crowds was the result of the interaction of three main elements: multisource pressure, state mutations and management responses. A series of factors interact and result in accidents, and the lack of a management response or a low-quality management response is the root cause of such accidents. A high-quality management response is a basic safety precaution for highly aggregated tourist crowds. Therefore, forming a virtuous circle of multisource pressure, state mutations and management responses is an effective path for coping with accidents.

## Introduction

With the growth of populations and the worldwide development of urbanization, the occurrence of crowd phenomena is increasingly frequent in large cities [1,2]. There is a wide range of crowd phenomena [3], including protests and demonstrations [4], crowding [5], football crowds [6], religious crowds [7], festival mass gatherings [8], and tourism crowding [9,10]. However, as the tourist population increases, highly aggregated tourist crowds (HATCs) appear more frequently. In China, under the catalysis of special time periods, HATCs are likely to appear in specific places. For example, tourists gather together and form HATCs in cable car stations, transfer centers and ticket offices in the popular tourist spots during the Golden Week. However, because of the high density and complexity of the crowd, it is easy for emergencies to occur within the crowds during mass events [11,12], including trampling accidents [13], such as the Shanghai stampede on the bund and the Love Parade disaster, which resulted in injuries and deaths [14]. Obviously, it is a difficult and important task for organizers to manage crowds [15,16]. Therefore, this paper focuses on the safety issues of HATCs due to their high potential risk, which will be useful for the management of HATCs.

Currently, research has been essential in studying the safety of dense crowds, but there has been comparatively less research concerning the safety of HATCs. Many studies on dense crowds have focused on the influencing factors affecting the safety of crowds, such as the crowd density and the crowd behavior. The density of a crowd is the root cause of accidents [17]. As the density of a crowd increases, the normal order of the crowd will gradually decrease [18]. The higher the density of the crowd is, the higher the potential risk of accidents [19–21]. Therefore, crowd safety is an important research issue [3]. Crowds with a density higher than 3–4 persons per square meter must be avoided [22]. In addition, the crowd density should be controlled so that it remains under 5 persons per square meter [1]. Therefore, many researchers regard 5 persons per square meter as the critical density [19,23]. If the density of a crowd is higher than 5 persons per square meter, it is dangerous for the crowd and is also likely to cause an accident. Therefore, much effort has been focused on estimating the density of crowds [24]. There are many methods to estimate the density of crowds, such as developing a system that uses wireless sensors and k-means clustering to estimate the density of crowds [25] or using a wearable electronic device to detect the crowd density [23].

However, the complex behavior of crowds is another root cause of accidents [26]. Because of the importance of crowd behavior to crowd safety [2], the research on crowd behavior has received much attention. Generally, the research on crowd behavior has focused on two major aspects: crowd behavior detection techniques and the analysis of crowd behavior in different situations. Many researchers have suggested using different techniques to detect crowd behavior, such as using video cameras[7,21,27], a tracklet-based model[28], a hybrid tracking model and a GSLM-based neural network[29], the particle entropy[30], and energy models[31], an Air Patrol Robot[32], an entropic path-integral model[33,34], and so on. In addition, a few researchers have analyzed the crowd behavior under different circumstances, such as during an earthquake evacuation[35], in a t-shaped channel[36], in a football stadium[37] and during high-stress evacuations[38].

An HATC is a special type of crowd phenomenon. With the normalization and popularization of tourism, HATCs appear increasingly more frequent [10]. However, few studies have explored the safety of HATCs. The crew, environment, facilities, management, space-time and other factors affect the safety of HATCs [39,40]. In addition, some studies have focused on the management of the safety of HATCs, such as congestion mitigation strategies [41], dynamic adjustments of travelling routes [42], and emergency evacuation strategies [43].

Accordingly, even though there have been a few studies that focused on the safety of HATCs, the safety of HATCs has not yet received adequate attention. According to the analysis of the literature, there are some research opportunities. First, there are not many comprehensive analyses of HATC accidents. The comprehensive analysis of crowd accidents would be helpful for forming a safety management strategy for crowds [1], such as analyzing the Love Parade disaster and proposing management suggestions [44]. It is regrettable that there has been a lack of analyses of multiple cases that could help us to form measures for the safety management of HATCs. Second, the occurrence mechanism and coping paths of HATCs accidents are not clear. It is necessary for managing crowds to determine the interactions of the influencing factors of crowd accidents [20].

According to the research opportunities, this paper will conduct a comprehensive analysis of HATC accidents. The objectives of this paper are as follows: 1) to comprehensively analyze and explore the factors influencing the safety of HATCs; 2) to determine how these influencing factors interact with each other and to investigate the occurrence mechanism of HATC accidents; and 3) to address the problem how to cope with HATC accidents and find the coping paths of HATC accidents.

HATCs are becoming increasingly more frequent. However, previous studies have focused on dense crowds and have not paid enough attention to HATCs, especially the safety of HATCs. Furthermore, while previous studies have paid much attention to how to use technology to detect crowd behavior in different situations, they have paid little attention to why crowd accidents occur and how to deal with them. Therefore, this paper focused on the causes of HATC safety accidents and how to address them. Considering the objectives of this study, this work makes significant theoretical contributions and is particularly valuable in two respects. Theoretically, this paper focuses on special crowd phenomena and explores the safety of HATCs, which can enrich the HATC research. Specifically, this paper determines the reasons why HATCs are insecure and forms a safe operation mechanism for HATCs. Meanwhile, this paper answered why HATC accidents occur and how to cope with them, which can fill the research gaps regarding the occurrence mechanism and coping paths of HATC accidents. Practically, this paper reveals the occurrence mechanism and coping paths of HATC accidents, which can provide references and enlightenment for strengthening the safety management of HATCs.

In the following section, this paper will introduce the materials and methods that we used to analyze these cases. Then, we will use system dynamics to analyze the interaction among the influencing factors under different situations and refine the occurrence mechanism of HATC accidents. With that, this paper will explore how to cope with HATC accidents and establish the coping path for such accidents. Last but not least, this paper will conclude with a discussion and future research prospects.

## Materials and methods

### Materials

Analyzing typical cases is an important way to research the safety of HATCs. Social media and web data analysis play important roles in crowd management[45]. Therefore, this paper used Baidu News and Sina Weibo to search for typical cases regarding the safety of HATCs for the following reasons. Baidu News is the largest Chinese news platform that was launched by China’s largest search engine Baidu [46–48], which can ensure the collection of a sufficient number of cases. Baidu News retains all the news since July 2003, which ensures that we can collect enough information. In addition, Sina Weibo is the most popular microblogging service in China with a total number of users that is close to the number of Twitter users [49]. Sina Weibo records what people see, hear, and experience in the self-organized platform and also provides a platform for data mining [50].

We used ten kinds of keywords, such as ‘Scenic + congestion’ and ‘Tourists + blowout’, to search for and collect the cases. In addition, this paper proposes five principles of case selection. First, the time period for the cases that were collected from Baidu News ranges from November 4, 2003 (Baidu News was launched on November 4, 2003) to May 31, 2017. Meanwhile, this paper collected cases from Sina Weibo from August 14, 2009 (the day when Sina Weibo was launched) to May 31, 2017. Second, we localized the cases in mainland China, including 22 provinces, 4 municipalities and 5 autonomous regions. Third, the cases that were collected from Baidu News were taken from the online version of published, local government official websites, professional news sites, and authoritative websites (including mainstream social media and websites in China, such as China’s mainstream media: Sina, Souhu, NetEase, www.toutiao.com, www.gmw.cn, people.cn, ifeng.com, etc.). Fourth, the cases that were collected from Baidu News and Sina Weibo were required to be accompanied by pictures or videos, which can help us to intuitively determine whether the case falls within the HATC category based on the visual information. In this paper, we treated the crowds whose density was higher than 3 persons per square meter and where more than 50 people were gathered together as HATCs. HATCs were mainly identified and defined according to two patterns. In this paper, whether a crowd was an HATC or not was judged mainly based on whether the news directly mentioned the density and population of the crowd. The second estimates whether the number and density of the crowd met the HATC standard according to the scene’s pictures and video. Fifthly, the text of the collected cases was required to be more than 200 words, which can ensure that the cases contain enough information. According to the above principles, this study collected 264 cases. Among these 264 cases, 158 cases came from Baidu News, and 106 cases originated from Sina Weibo. Our data collection method complied with the terms and conditions for the websites from which we collected data (Sina Weibo and Baidu News). Besides, all the personal information of tourists involved in the cases is protected.

These 264 cases in this study involved different spatial types. From a macro perspective, the accidents took place in multiple scenarios, including mountainous places (56.44%), folk custom places (8.71%), historical places and sites (7.20%), rivers and lakes attraction (6.44%), theme parks (4.55%), ancient blocks (4.17%), coastal islands attraction (3.79%), public areas (3.03%), ancient sites (3.03%), classical gardens (1.14%), biological landscape places (1.14%), other places (0.38%) and other places than these 12 classes. From the micro perspective, the accidents occurred in three kinds of micro spaces, including key nodes (66.67%, consisting of scenic site entrances, funiculars, floating docks, scenic spot ticket windows, hot spots, etc.), key sections (18.94%, consisting of swim trails, plank roads, glass plank roads, town streets, rivers, etc.), and important areas (14.39%, consisting of squares, parking lots, ancient blocks, temple fairs, etc.).

### Method

System dynamics (SD) as a system analysis method that was founded by Jay Forrester for industrial dynamic management [51]. SD is grounded on the theory of nonlinear dynamics and feedback control, and it also combines the knowledge of other social sciences, such as economics and social psychology, to analyze the complex behaviors of systems [52]. It has been found that SD is a useful methodology to analyze complex systems and behaviors using techniques such as influence and causal loop diagrams and computer simulations [51,53,54]. Especially, the causal loop modeling of SD focuses on the feedback among the variables in a system [53,55–57], which can effectively explore the interactions among the various factors and determine the causal relationship among the elements [58,59].

The occurrence of accidents is the result of the interactions of multiple factors. It is necessary to figure out the interactions among the various factors influencing the accidents when we want to explore the occurrence mechanism of accidents[60]. SD is known for its ability to unravel complexity[53] and explore the interactions among the various factors. Because of the unique benefits of SD, this method has been widely used in accident and disaster research, such as that of the Westray mine disaster [61], organizational accidents[55,62], ship accidents [63] and aircraft accidents [52].

A crowd is a living system [64]. Furthermore, the root cause of crowd accidents lies in the failure of the system [44]. Thus, we can analyze HATC accidents from a systematic perspective. Furthermore, the occurrence of HATC accidents is the result of the interactions of multiple factors [65]. Therefore, this paper attempts to analyze the occurrence mechanism of HATC accidents using system dynamics, especially the causal loop modeling of SD.

## Results

### The factors influencing the safety of HATCs

This paper comprehensively analyzed cases and refined the factors influencing the safety of HATCs. Subsequently, grounded theory was applied to identify the factors that influence the security of HATCs. The following process was applied. Open coding analysis was performed based on the following logical analytical procedure: Define the Phenomenon, Develop the Concept, and Extract the Category. According to the analysis of the open coding of grounded theory, by defining the phenomenon (the authors found and defined 216 phenomenon), conceptualization (according to the 216 phenomenon, the authors identified 138 preliminary concepts), standardization (the authors extracted 68 normative concepts by reanalyzing the 138 preliminary concepts) and categorization (the authors concluded 26 influencing factors after combining and reanalyzing the 68 normative concepts), the authors gradually and finally extracted the 26 influencing factors [65], which are shown in Table 1.

**Table 1:** The factors influencing the safety of HATCs.

Factor	Context	Factor	Context
F1	Catalyst of a special time period	F14	Emergency plan response
F2	Insufficient warning for tourists	F15	Management response for tourists
F3	Pressure of tourist gatherings	F16	Advance management response for tourists
F4	Stimulation of attractive elements	F17	Strengthened management response
F5	Pressure produced by tourists	F18	Insufficient management response for tourists
F6	Environmental pressure of traveling	F19	Insufficient joint response
F7	Service pressure	F20	Insufficient management response
F8	Failure of the service’s function	F21	Lack of an emergency plan
F9	The psychological status of tourists	F22	Lack of an advanced management response
F10	The physical status of tourists	F23	Lack of a management response for tourists
F11	The state of tourist behavior	F24	Lack of a management response
F12	Tourists’ complaints	F25	Proposing solutions
F13	The state of the order of the tourist crowd	F26	Joint response

The results revealed that there were 26 influencing factors that affected the security of HATCs, including the tourist flow pressure, tourist behavior status, and enhanced management response. The application of a typical grounded theory analytical framework, namely, ‘causal condition-phenomenon-context- mediating condition-action/interaction strategy-result’, revealed that the following five factors influenced the security of HATCs: multiple source pressure, state variations, high-quality emergency management responses, poor emergency management responses, and emergency management response losses.

Different types of pressure are one of the causes of crowd accidents [66]. According to Table 1, this paper found that there were many types of pressures that HATCs faced, such as F3 (pressure of tourist gatherings); F6 (environmental pressure of traveling); and F7 (service pressure). These different types of pressures are combined and referred to as multisource pressures. Thus, the first main category is multisource pressure, and it includes F1, F2, F3, F4, F5, F6, and F7.

The sudden changes of the state are often associated with crowd accidents [34]. According to the analysis of the HATC accident cases, there were many sudden state changes, such as sudden changes of the service state, sudden changes of the psychological status of tourists, sudden changes of the state of tourist behavior, and so on. Considering the state changes during the accidents, this paper defined the special phenomenon as a state mutation. The state mutation referred to a dramatic and sudden changes of the state, including F8 (failure of the service’s function), F9 (the psychological status of tourists), F10 (the physical status of tourists), F11 (the state of tourist behavior), F12 (tourists’ complaints), and F13 (the state of the order of the tourist crowd).

However, whether an accident happened or not is directly affected by the quality of the management response of an organization [52,54]. According to the 264 cases, this paper divided the emergency management responses into three aspects: the lack of an emergency management response, a low quality emergency management response and a high-quality emergency management response. All of the cases of the lack of an emergency management response, a poor emergency management response and a high-quality emergency management response are concluded based on the statement of the cases. According to the factors affecting the safety of HATCs that are shown in Table 1, this paper identified low quality emergency management responses by referring to negative emergency responses to multisource pressures and state mutations, including F18 (insufficient management response for tourists), F19 (insufficient joint response) and F20 (insufficient management response [45]). However, high-quality emergency management responses refer to the positive responses of organizations to multisource pressures and state mutation, including F14 (response of the emergency plan), F15 (management response for tourists), F16 (advanced management response for tourists), F17 (strengthened management response), F25 (proposing solutions) and F26 (joint response). When facing multisource pressures and state mutations, if the organization did not conduct any emergency response, we defined it as the lack of an emergency management response, including F21 (lack of an emergency plan), F22 (lack of an advanced management response), F23 (lack of a management response for tourists), and F24 (lack of a management response).

### The operational mechanism of HATCs in a safe state

Based on the 264 accident cases, this paper extracted and filtered the relationships among the 26 factors that are shown in Table 1 by analyzing the stated related factors in the text of the cases. A typical statement of the related factors in the cases and the relationships among these factors in a safe state are shown in Table 2.

**Table 2:** The typical statements of the related factors and their interactive relationships of operational mechanism of HATCs in a safe state.

interactive relationships between factors	The typical statements of the related factors
F3→+F8	Due to the large number of tourists that were gathering (F3, Pressure of tourist gatherings), the tourist buses must stop two kilometers away from the ropeway, and the tourists walked to the ropeway
F3→+F11	Nearly a thousand tourists have been stuck here for approximately 30 minutes (F3, pressure of tourist gatherings), and impatient tourists clambered up the wall to find another way (F11, the state of tourist behavior).
…….	……

**Fig 1:**
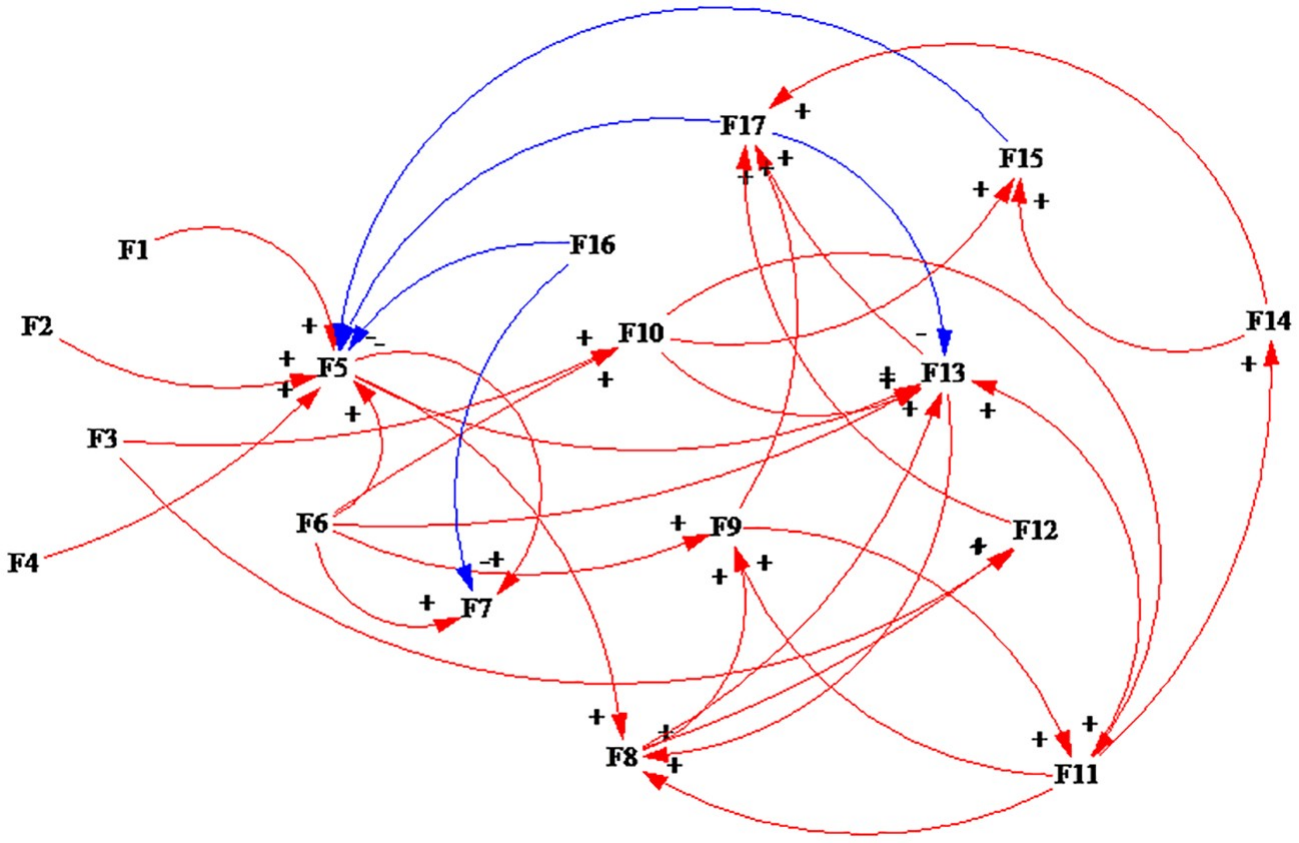
The interaction among the elements of the HATCs in a safe state.

After analyzing the interactions of the factors in safe states, we used the Vensim PLE 7.0 software [54,67] to draw the causal loop diagram of the interaction of the factors influencing the safety of HATCs, which is shown in Fig 1 (“+” means a positive effect and “-” means a negative effect). These elements interact with each other and form 81 causal loops. The typical interaction among the factors is shown as follows.

The interactive relationship of the three factors is as follows: F5 positively affects F8, F8 positively affects F15 and F15 negatively affects F5. In addition, F5 positively affects F11, F11 positively affects F17, and F17 negatively affects F5.

The interactive relationship among four elements is as follows: F5 positively affects F11, F11 positively affects F8, F8 positively affects F15, and F15 negatively affects F5. In addition, F5 positively affects F11, F11 positively affects F13, F13 positively affects F17, and F17 negatively affects F5.

The interactive relationship among five elements is as follows: F5 positively affects F9, F9 positively affects F11, F11 positively affects F8, F8 positively affects F17, and F17 negatively affects F5. F5 positively affects F9, F9 positively affects F11, F11 positively affects F14, F14 positively affects F17, and F17 negatively affects F5.

The interactive relationship among six elements is as follows: F5 positively affects F9, F9 positively affects F11, F11 positively affects F13, F13 positively affects F8, F8 positively affects F15, and F17 negatively affects F5. F5 positively affects F7, F7 positively affects F8, F8 positively affects F11, F11 positively affects F13, F13 positively affects F17, and F17 negatively affects F5.

The relationship among seven elements is as follows: F5 positively affects F13, F13 positively affects F9, F9 positively affects F11, F11 positively affects F14, F14 positively affects F17, and F17 negatively affects F5.

**Fig 2:**
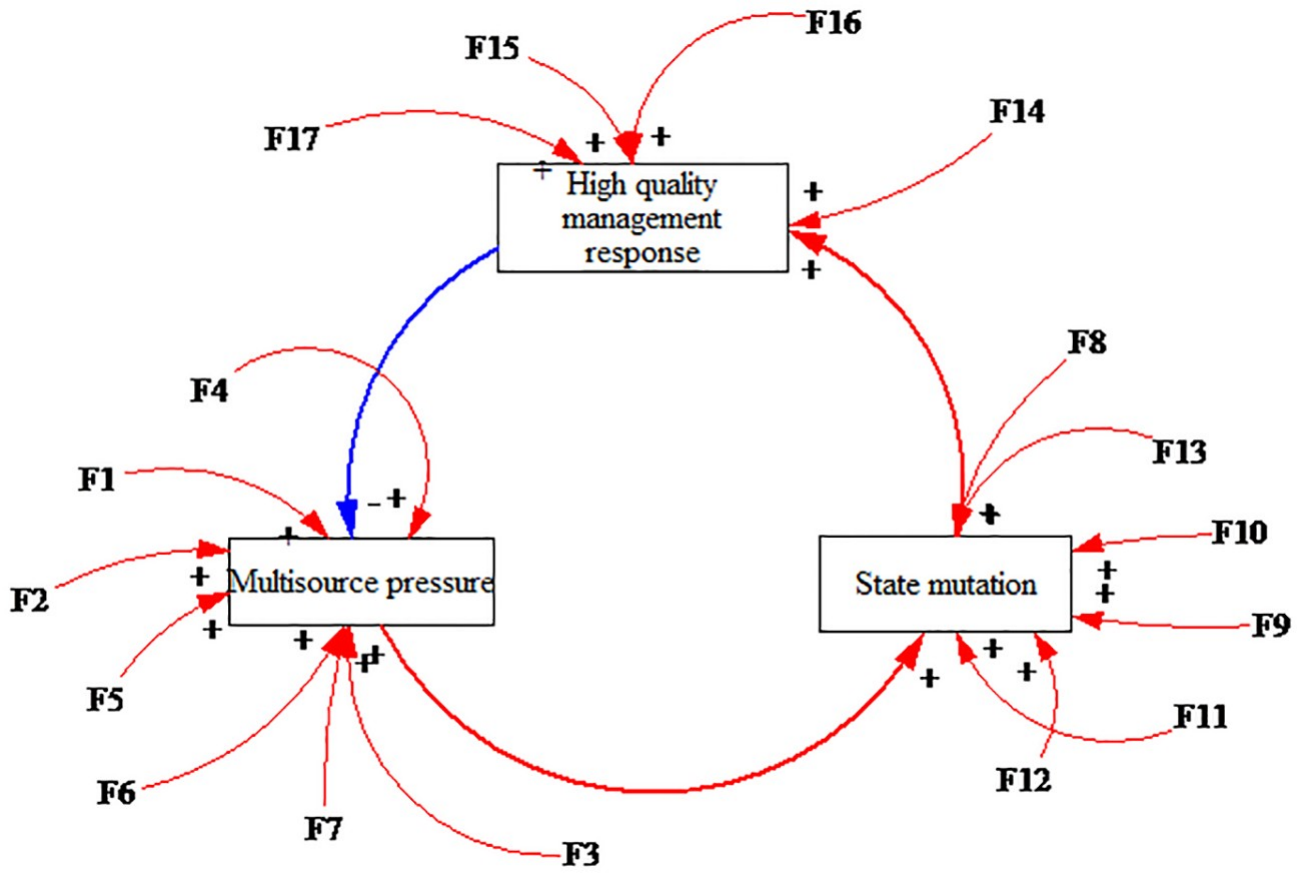
The operating mechanism of highly aggregated tourist crowds in a safe state.

This paper refined and proposed the operating mechanism that is shown in Fig 2 according to the interaction among the influencing factors in a safe state. Specifically, many kinds of substantial pressures appear in HATCs, which we refer to as multisource pressure [66,68]. The multisource pressure results in the state mutation of HATCs. If the organizer developed a high-quality management response, which could reduce the multisource pressure and state mutation, then the HATC would be in a safe state. It is the positive interaction among the multisource pressure, state mutation and high-quality management response that ensures the safety of an HATC.

### The occurrence mechanism of accidents

In accidents, the different factors resulted in different interactions among these factors. Therefore, this paper extracted and filtered the relationships among the 26 factors that are shown in Table 1 by analyzing the statement of the related factors of the text of the cases. The typical statements of the related factors in the cases and the relationships among these factors are shown in Table 3.

**Table 3:** The typical statements of the related factors and their interactive relationships of occurrence mechanism of accidents.

Interactive relationships between factors	A typical statement of the related factors
F18→+accident	However, the staff of the scenic spot is obviously not enough (F18, insufficient management response for tourists), and some tourists were retained (accident) at the top of the mountain before 10 am
F23→+accident	There was no staff to maintain the order of the tourists (F23, lack of a management response for tourists), and so the tourists were retained (accident)
…….	……

**Fig 3:**
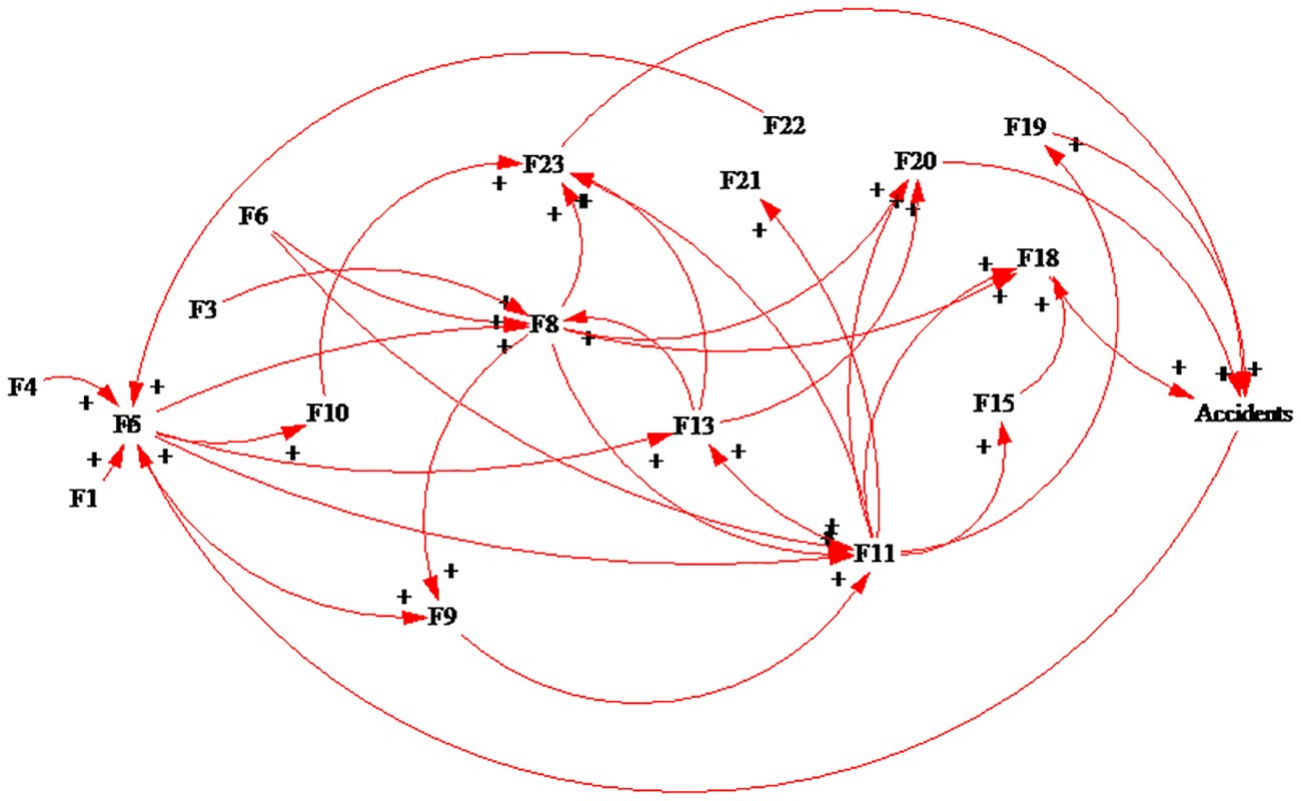
The interaction among the elements of HATCs in accidents.

After analyzing the interactions of the factors in accidents, we used the Vensim PLE 7.0 software to draw the causal loop diagram (shown in Fig 3) of the factors resulting in accidents in HATCs.

According to Fig 3, there are 17 influencing elements for accidents. These elements interact with each other and form 53 causal loops. The typical loops are as follows:

The relationship among three elements is that F5 positively affects F11, F11 positively affects F23 and results in accidents, and F23 has a positive effect on F5. F5 positively affects F8, F8 positively affects F18 and results in accidents, and F18 has a positive effect on F5.

The relationship among four elements is as follows: F5 positively affects F8, F8 positively affects F11, and F11 positively affects F19, which results in accidents. Then, the accidents positively affect F5, F5 positively affects F11, F11 positively affects F13, and F13 positively affects F23, which results in accidents. Then, the accidents positively affect F5.

The relationship among five elements is as follows: F5 positively affects F11, F11 positively affects F13, F13 positively affects F8, and F8 positively affects F23, which results in accidents. Then, the accidents have positive effects on F5. F5 positively affects F8, F8 positively affects F9, F9 positively affects F11, and F11 positively affects F19, which results in accidents. Then, the accidents positively affect F5.

The relationship among six elements is as follows: F5 positively affects F8, F8 positively affects F9, F9 positively affects F11, F11 positively affects F13, and F13 positively affects F18, which results in accidents. Then, the accidents positively affect F5. F5 positively affects F8, F8 positively affects F9, F9 positively affects F11, F11 positively affects F13, and F13 positively affects F23, which results in accidents. The accidents have positive effects on F5.

The relationship among seven elements is as follows: F5 positively affects F13, F13 positively affects F8, F8 positively affects F9, F9 positively affects F11, F11 positively affects F15, and F15 positively affects F18, which results in accidents. The accidents have positive effects on F5.

It should be noted that when the management of the HATCs fails to respond or responds insufficiently, the risk of accidents may be aggravated, or the situation may even lead to accidents.

**Fig 4:**
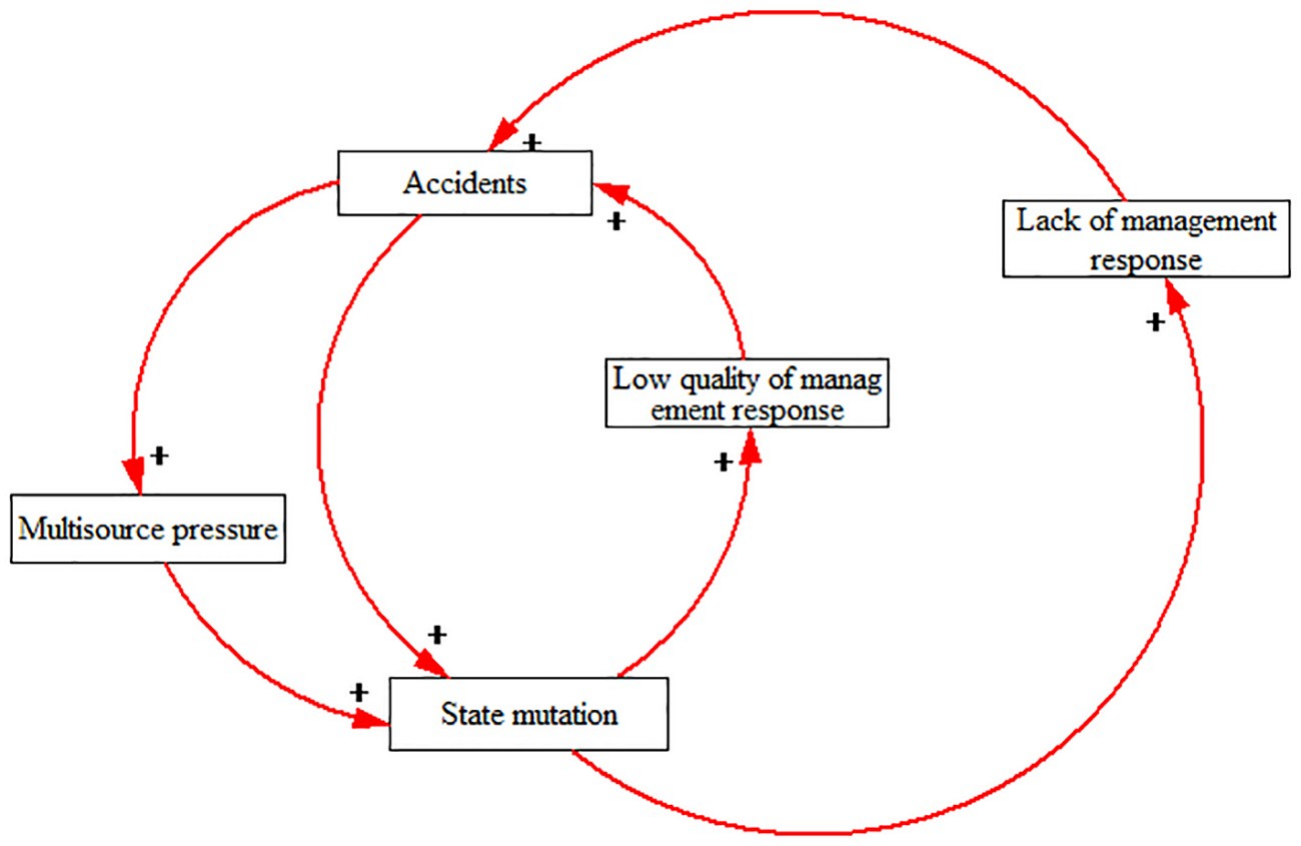
The occurrence mechanism of HATC accidents.

According to the interactive relationship among these elements in accident situations, this paper refined and proposed the occurrence mechanism (shown in Fig 4) of HATC accidents.

Specifically, accidents are prone to occur in HATCs under two situations: First, when the management response cannot effectively manage the state mutation and reduce the multisource pressure appearing in HATCs, this means that there is a poor management response. Second, when there is a lack of management response to multisource pressure and state mutation, accidents are likely to occur.

## The analysis of the coping paths of HATC accidents

### The coping paths of the accidents

After the accidents happen, how do these factors interact with each other and how can one cope with these accidents? To explore the coping paths of accidents, this paper extracted and filtered the relationships among the 26 factors that are shown in Table 1 by analyzing the statements of the related factors in the text of the cases. The typical statements of the related factors in the cases and the relationships among these factors are shown in Table 4.

**Table 4:** The typical statements of the related factors and their interactive relationships of coping paths of the accidents.

Interactive relationships between factors	The typical statements of the related factors
accident→+F14→+F17	As tourists descended the mountain at the same time, a large number of tourists gathered together and were retained at night (accident). The tour bus company launched emergency plans to evacuate the tourists (F14, response of the emergency plan), such as increasing the number of buses (F17, strengthened management response).
……	……

**Fig 5:**
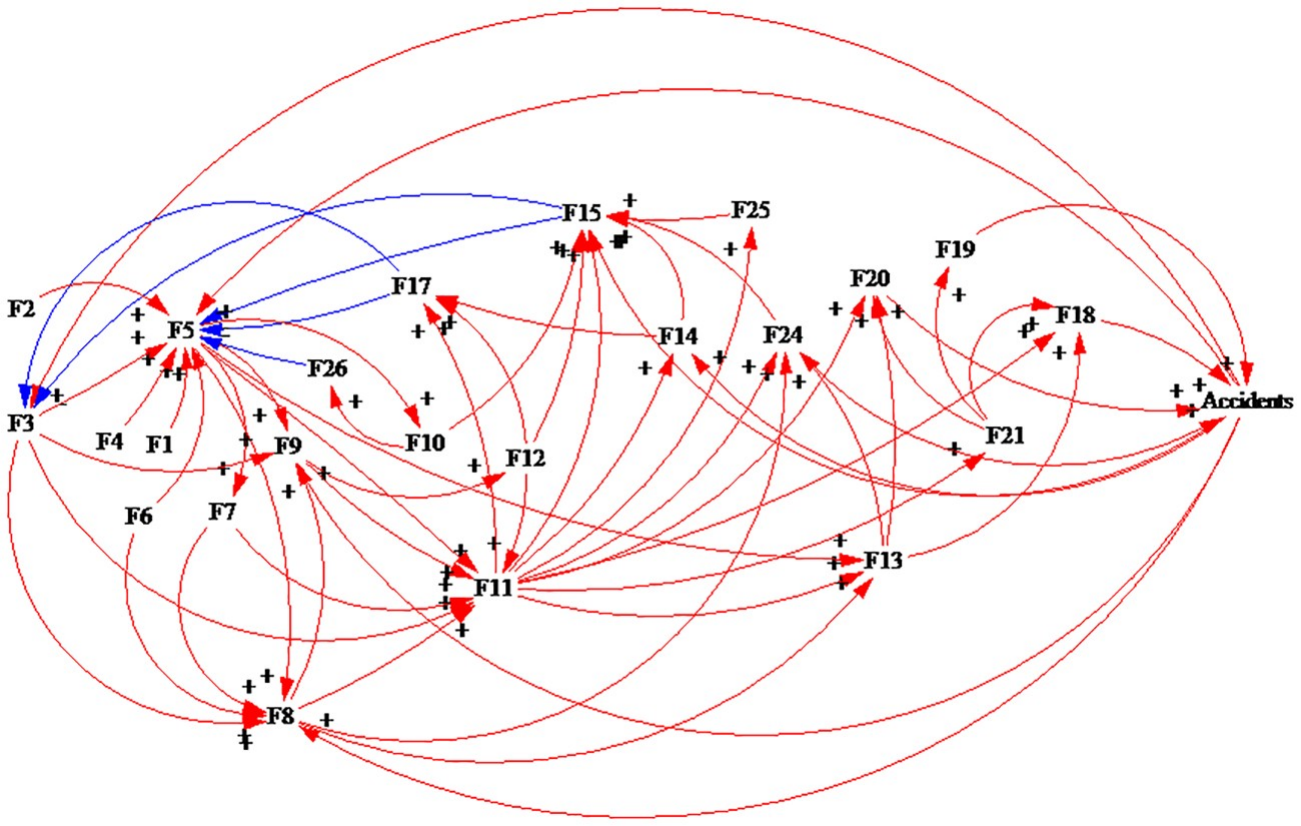
The coping paths of HATC accidents.

After analyzing the interactions of the factors after the accidents happened, this paper proposes the coping paths of HATC accidents. The causal loop diagram of the coping paths of the accidents is shown as Fig 5. These elements interact with each other and form 15 causal loops. The typical loops include the following.

Typical loop 1: F1 positively affects F5, F5 positively affects F7, F7 positively affects F11, and F11 positively affects F23, which results in accidents. The accidents have a positive effect on F15, which has a negative effect on F5.

Typical loop 2: F4 positively affects F5, F5 positively affects F7, and F7 positively affects F8, which results in accidents. The accidents positively affect F14, F14 positively affects F17, and F17 negatively affects F5.

Typical loop 3: F1 positively affects F5, F5 positively affects F13, and F13 positively affects F20, which results in accidents. The accidents have a positive effect on F11, F11 positively affects F9, F9 positively affects F15, and F15 has a negative effect on F5.

Typical loop 4: F5 positively affects F11, and F11 positively affects F24, which results in accidents. The accidents positively affect F9, F9 positively affects F11, F11 positively affects F15, and F15 negatively affects F5.

Typical loop 5: F5 positively affects F13, and F13 positively affects F24, which results in accidents. The accidents have a positive effect on F8, F8 positively affects F9, F9 positively affects F11, F11 positively affects F15, and F15 has a negative effect on F5.

According to Fig 5, after the occurrence of HATC accidents, the multisource pressure and state mutation will be aggravated. Therefore, when organizers make a management response to an accident, they must take any new changes into account and reinforce the original management response.

### The coping mechanism of HATC accidents

**Fig 6:**
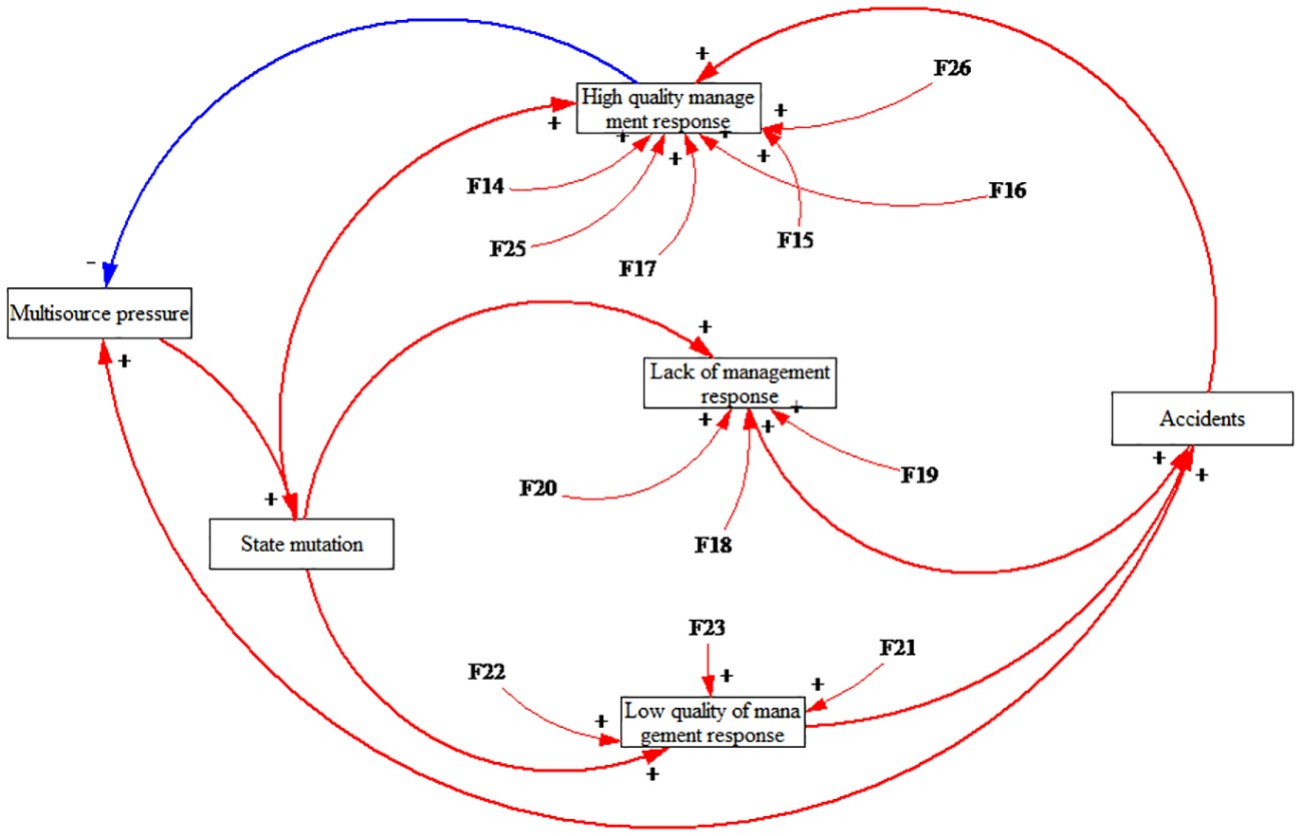
The response mechanism of HATC accidents.

Based on the coping paths of the accidents, this paper refined and proposed the coping mechanism (shown in Fig 6) of HATC accidents.

When the HATC accidents appeared, the first principle of the response to the accidents was to adjust the management response and strengthen the management response measures to adapt to the new changes in the multisource pressure subsystem and the state mutation subsystem resulting from the accidents. Therefore, we can follow the subsequent path to cope with the accidents: Multisource pressure positively affected the state mutation, and the state mutation positively affected the poor management response / lack of a management response, which resulted in accidents. Accidents had positive effects on the multisource pressure, the multisource pressure positively affected the state mutation, and the state mutation positively affected the high-quality management response, which negatively affected the multisource pressure. Once the accidents occurred, if the organizer could form a high-quality management response that could result in mutual coordination and mutual coupling with the multisource pressure and the state mutation, HATCs would return to a safe state.

## Conclusion and prospects

### Conclusion

According to the comprehensive analysis, this paper found that the different states of HATCs are the results of the interaction of three main elements: multisource pressure, state mutations and management responses. The safe state of HATCs is the result of positive interactions among the multisource pressure, state mutations and a high-quality management response. The lack of a management response or a low-quality management response is the root cause of accidents because the lack of a management response or a low-quality management response cannot effectively reduce multisource pressure and improve the state mutation. The coping path of HATC accidents is as follows: multisource pressure positively affects the state mutation, and the state mutation positively affects the poor management response / lack of a management response, which results in accidents. Accidents have positive effects on the multisource pressure, the multisource pressure positively affects the state mutation, and the state mutation positively affects a high-quality management response, which negatively affects the multisource pressure.

This paper proposed some suggestions for the management of HATCs based on the occurrence mechanism and coping paths. First, the management response for HATCs should be strengthened. A high-quality management response is the basic foundation for the safety of HATCs. Therefore, organizers should strengthen the management response and form high-quality management responses. Second, this process attempts to form a virtuous circle of multisource pressure, state mutations and management responses. Organizers should dynamically assess the multisource pressure and state mutation targets to propose a high-quality management response and form a virtuous circle of the three main elements.

### Limitations and further research

This paper not only comprehensively analyzed the HATC accidents but also determined the occurrence mechanism and coping paths of HATC accidents. Inevitably, there are some limitations in this paper. First, this paper revealed the occurrence mechanism and coping paths of HATC accidents. We focused on the common factors influencing the occurrence of accidents and accident handling; however, the differentiation produced by different spatial situations has not been addressed in this study. In previous studies, many scholars have used spatial and temporal characteristics to explore the abnormal behaviors of crowds [69–71]. Therefore, it is necessary to strengthen the discussion of crowd behavior in different spaces in the future, and further study of different crowd management strategies in different spaces should be performed to determine effective management measures for HATCs. Second, this paper only qualitatively analyzed the occurrence mechanism and coping paths of HATCs accidents, which need to be empirically tested in the future. Furthermore, the safety assessment of dense crowds and the simulation of crowd behavior can be treated as a future research direction [26]. Crowd behavior was acknowledged as a complex phenomenon[72]. Thus, the simulation of crowd behavior is helpful for crowd management[73]. Therefore, we should focus on simulating crowds and developing targeted crowd management advice. In summary, future researchers can combine the occurrence mechanism of HATC accidents, construct a system dynamics model for HATCs, produce a simulation model for HATCs, dynamically assess the status of HATCs and develop an early warning mechanism for HATCs.

## Supporting information

S1 Dataset Data set. (XLS)

S1 Ethics Ethics statement. (PDF)

## References

[1] Zhan B, Monekosso DN, Remagnino P, Velastin SA, Xu L-Q (2008) Crowd analysis: a survey. Machine Vision and Applications 19: 345–357.

[2] Johansson A, Batty M, Hayashi K, Al Bar O, Marcozzi D, Memish Z. (2012) Crowd and environmental management during mass gatherings. The Lancet Infectious Diseases 12: 150–156. 10.1016/S1473-3099(11)70287-0 22252150

[3] Alnabulsi H, Drury J (2014) Social identification moderates the effect of crowd density on safety at the Hajj. Proc Natl Acad Sci USA 111: 9091–9096. 10.1073/pnas.1404953111 24927593 PMC4078860

[4] Reicher SD (1996) The Battle of Westminster’:Developing the social identity model of crowd behaviour in order to explain the initiation and development of collective conflict. European Journal of Social Psychology 26: 115–134.

[5] Novelli D, Drury J, Reicher S, Stott C (2013) Crowdedness mediates the effect of social identification on positive emotion in a crowd: A survey of two crowd events. PloS one 8: e78983 10.1371/journal.pone.0078983 24236079 PMC3827307

[6] Stott C, Adang O. L A., Schreiber M (2007) Variability in the collective behaviour of England fans at Euro2004 ‘Hooliganism’, public order policing and social change. European journal of social psychology 37: 75–100.

[7] Cassidy C, Hopkins N, Levine M, Pandey J, Reicher S, Singh P (2007) Social identity and collective behaviour: Some lessons from Indian research at the Magh Mela at Prayag. Psychological Studies-University of Calicut 52: 286.

[8] Neville F, Reicher S (2011) The experience of collective participation: Shared identity, relatedness, and emotionality. Contemporary Social Science 6: 377–396.

[9] Popp M (2012) Positive and Negative Urban Tourist Crowding: Florence, Italy. Tourism Geographies 14: 50–72.

[10] Shi B, Zhao J, Chen P-J (2017) Exploring urban tourism crowding in Shanghai via crowdsourcing geospatial data. Current Issues in Tourism 20: 1186–1209.

[11] Zachko O, Golovatyi R, Yevdokymova A (2017) Development of a simulation model of safety management in the projects for creating sites with mass gathering of people. Eastern-European Journal of Enterprise Technologies 86: 15–24.

[12] Khozium MO, Abuarafah AG, AbdRabou E (2012) A proposed computer-based system architecture for crowd management of pilgrims using thermography. Life Science Journal 9: 377–383.

[13] Mehdi M, Dirk H, Guy T (2011) How simple rules determine pedestrian behavior and crowd disasters. Proceedings of the National Academy of Sciences 108: 6884–6888. 10.1073/pnas.1016507108 PMC3084058 21502518

[14] Simon A (2017) Crowd planning for public safety. Perspectives in Public Health 137: 25–28. 10.1177/1757913916681265 28074698

[15] Zhang XL, Weng WG, Yuan HY, Chen JG (2013) Empirical study of a unidirectional dense crowd during a real mass event. Physica A: Statistical Mechanics and its Applications 392: 2781–2791.

[16] Pin SC, Haron F, Sarmady S, Talib AZ, Khader AT (2011) Applying TRIZ principles in crowd management. Safety Science 49: 286–291.

[17] Saad A, H.,Algadhi, Hani S, Mahmassani (1990) Modelling Crowd Behavior and Movement Application to Makkah Pilgrimage. Transportation and traffic theory: 59–78.

[18] Shelby B, Vaske J, J. (1991) Using Normative Data to Develop Evaluative Standards for Resource Management: A Comment on Three Recent Papers. Journal of Leisure Research 23: 173.

[19] Lee RS, Hughes RL (2005) Exploring trampling and crushing in a crowd. Journal of transportation engineering 131: 575–582.

[20] Lee RS, Hughes RL (2006) Prediction of human crowd pressures. Accid Anal Prev 38: 712–722. 10.1016/j.aap.2006.01.001 16487473

[21] Karpagavalli P, Ramprasad AV (2013) Estimating the density of the people and counting the number of people in a crowd environment for human safety. International conference on Communication and Signal Processing. pp. 663–667.

[22] Anders J, Dirk H (2008) From Crowd Dynamics to Crowd Safety:A Video-Based Analysis. Advances in Complex Systems 11: 497–527.

[23] Angela TYH, Viswanathan V, Lees M, Cai W (2014) Analysing the Effectiveness of Wearable Wireless Sensors in Controlling Crowd Disasters. Procedia Computer Science 29: 1590–1599.

[24] Rahmalan H, Nixon MS, Carter JN (2006) On crowd density estimation for surveillance. IET Conference on Crime and Security. pp. 540–545.

[25] Yuan Y, Qiu C, Xi W, Zhao J (2011) Crowd Density Estimation Using Wireless Sensor Networks. 2011 Seventh International Conference on Mobile Ad-hoc and Sensor Networks. pp. 138–145.

[26] Shi X, Ye Z, Shiwakoti N, Tang D, Wang C, Wang W (2016) Empirical investigation on safety constraints of merging pedestrian crowd through macroscopic and microscopic analysis. Accid Anal Prev 95: 405–416. 10.1016/j.aap.2015.10.009 26519346

[27] Alfredo C, Emanuele Z (2006) Analysis of Jumping Crowd on Stadium Stands Through Image Processing to Security Purposes. IEEE International Workshop on Measurement Systems for Homeland Security, Contraband Detection and Personal Safety: 56–61.

[28] Rabiee H, Mousavi H, Nabi M, Ravanbakhsh M (2018) Detection and localization of crowd behavior using a novel tracklet-based model. International Journal of Machine Learning and Cybernetics 9: 1999–2010.

[29] Manoj K, Charul B (2017) Hybrid tracking model and GSLM based neural network for crowd behavior recognition. Journal of Central South University 24: 2071–2081.

[30] Gu X, Cui J, Zhu Q (2014) Abnormal crowd behavior detection by using the particle entropy. Optik 125: 3428–3433.

[31] Xiong G, Cheng J, Wu X, Chen Y-L, Ou Y, Xu Y (2012) An energy model approach to people counting for abnormal crowd behavior detection. Neurocomputing 83: 121–135.

[32] Zhao H, Wang S, Xu S, Zhang Y, Sugisaka M (2017) A Method of Detecting Abnormal Crowd Behavior Events Applied in Air Patrol Robot. Journal of Robotics, Networking and Artificial Life 4: 77–80.

[33] Ivancevic VG, Reid DJ, Aidman EV (2010) Crowd behavior dynamics: entropic path-integral model. Nonlinear Dynamics 59: 351–373.

[34] Zhao Y, Yuan M, qi, Su G, feng, Chen T (2015) Crowd Security Detection based on Entropy Model. the ISCRAM 2015 Conference. pp. 24–33.

[35] Liu T, Liu Z, Ma M, Chen T, Liu C, et al (2018) 3D visual simulation of individual and crowd behavior in earthquake evacuation. Simulation 95: 65–81.

[36] Yuan Z, Guo R, Tang S, He B, Bian L, Li Y (2019) Simulation of the Separating Crowd Behavior in a T-Shaped Channel Based on the Social Force Model. IEEE Access: 13668–13682.

[37] Sikora W, Malinowski J (2013) Crowd Behavior as an Example of the Evolution of a Complex System—Evacuation Models Proposal Based on the Symmetry Analysis Approach. Acta Physica Polonica A 124: 1005–1012.

[38] Moussaıd M, Kapadia M, Thrash T, Sumner RW, Gross M, Helbing D, et al (2016) Crowd behaviour during high-stress evacuations in an immersive virtual environment. Journal of The Royal Society Interface 13. 10.1098/rsif.2016.0414 PMC5046946 27605166

[39] Yin J, Zheng X, Dong B, Jiao N (2016) Tourists crowded places: concept, characteristics, risks and research topics. Journal of Chongqing Technology and Business University(Social Science Edition): 34–41.

[40] Yin J, Zheng X (2017) The Research on the Safety Risk of Tourists Crowded Places Which is Based on the Optimal Dimension Analysis. Science Economy Society: 76–82.

[41] Wang Y, Li Q, Chen J (2011) Simulation of Congestion Alleviating Strategies in Sightseeing Public Places. China Safety Science Journal: 27–32.

[42] Ren J, Zheng W (2013) Dynamic real-time scheduling simulation of tourist routes in the peak tourist area. Statistics and Decision: 42–45.

[43] Zhang Y, Chen M (2014) Research on rapid evacuation method of tourist in crowded areas. Computer Simulation: 432–435.

[44] Helbing D, Mukerji P (2012) Crowd disasters as systemic failures:analysis of the Love Parade disaster. EPJ Data Science 1: 1–40.

[45] Sharma D, Bhondekar AP, Shukla AK, Ghanshyam C (2018) A review on technological advancements in crowd management. Journal of Ambient Intelligence and Humanized Computing 9: 485–495.

[46] Shen D, Zhang W, Xiong X, Li X, Zhang Y (2016) Trading and non-trading period Internet information flow and intraday return volatility. Physica A: Statistical Mechanics and its Applications 451: 519–524.

[47] Song P, Zhang C, Xu Y, Huang L (2010) Brand extension of online technology products: Evidence from search engine to virtual communities and online news. Decision Support Systems 49: 91–99.

[48] Wang J, Fu Z, Cheng J, Ma F (2010) The Measurement of Relative Recall with Weights: a Perspective of User Feedback. Journal of Computers 5.

[49] Qiu L, Lu J, Yang S, Qu W, Zhu T (2015) What does your selfie say about you? Computers in Human Behavior 52: 443–449.

[50] Xu Z, Zhang H, Sugumaran V, Choo K-KR, Mei L, Zhu Y (2016) Participatory sensing-based semantic and spatial analysis of urban emergency events using mobile social media. EURASIP Journal on Wireless Communications and Networking: 44.

[51] Forrester JW (1961) Industrial Dynamics. Cambridge: MIT Press.

[52] Lu Y, Zhang S-G, Hao L, Huangfu H-Y, Sheng H (2016) System dynamics modeling of the safety evolution of blended-wing-body subscale demonstrator flight testing. Safety Science 89: 219–230.

[53] Goh YM, Love PED (2012) Methodological application of system dynamics for evaluating traffic safety policy. Safety Science 50: 1594–1605.

[54] Bouloiz H, Garbolino E, Tkiouat M, Guarnieri F (2013) A system dynamics model for behavioral analysis of safety conditions in a chemical storage unit. Safety Science 58: 32–40.

[55] Goh YM, Love PED, Brown H, Spickett J (2012) Organizational Accidents: A Systemic Model of Production versus Protection. Journal of Management Studies 49: 52–76.

[56] Zhao B, Tang T, Ning B (2017) System dynamics approach for modelling the variation of organizational factors for risk control in automatic metro. Safety Science 94: 128–142.

[57] Shin M, Lee HS, Park M, Moon M, Han S (2014) A system dynamics approach for modeling construction workers’ safety attitudes and behaviors. Accid Anal Prev 68: 95–105. 10.1016/j.aap.2013.09.019 24268437

[58] Lane DC, Monefeldt C, Rosenhead JV (2016) Looking in the wrong place for healthcare improvements:A system dynamics study of an accident and emergency department. Operational Research for Emergency Planning in Healthcare 2: 92–121.

[59] Garbolino E, Chery JP, Guarnieri F (2016) A Simplified Approach to Risk Assessment Based on System Dynamics:An Industrial Case Study. Risk Analysis 36: 16–29. 10.1111/risa.12534 26765309

[60] Davies JC, Stevens G, Manningc DP (1998) Understanding accident mechanisms: an analysis of the components of 2516 accidents collected in a MAIM database. Safety Science 29: 25–58.

[61] Cooke DL (2003) A system dynamics analysis of the Westray mine disaster. System Dynamic Review 19: 139–166.

[62] Marais K, Saleh JH, Leveson NG (2006) Archetypes for organizational safety. Safety Science 44: 565–582.

[63] Jeon JW, Wang Y, Yeo GT (2016) Ship Safety Policy Recommendations for Korea: Application of System Dynamics. The Asian Journal of Shipping and Logistics 32: 73–79.

[64] Bellomo N, Clarke D, Gibelli L, Townsend P, Vreugdenhil B, J (2016) Crowd dynamics and safety Reply to comments on “Human behaviours in evacuation crowd dynamics: From modelling to “big data” toward crisis management”. Physics of Life Review 18: 55–65. 10.1016/j.plrev.2016.08.014 27639539

[65] Yin J, Zheng X, min (2018) Application of Grounded Theory to Identify Factors Influencing the Security of Highly Aggregated Tourist Crowds and their Implementation Paths. Tourism Tribune 33: 133–144.

[66] Dirk H, Anders J, Al-Abideen Habib Z (2007) The Dynamics of Crowd Disasters: An Empirical Study. Physical review E 75: 1–7. 10.1103/PhysRevE.75.046109 17500963

[67] Eberlein Robert L, Peterson David W (1992) Understanding models with Vensim. European Journal of Operational Research 59: 216–219.

[68] Alaska YA, Aldawas AD, Aljerian NA, Memish ZA, Suner S (2017) The impact of crowd control measures on the occurrence of stampedes during Mass Gatherings: The Hajj experience. Travel Med Infect Dis 15: 67–70. 10.1016/j.tmaid.2016.09.002 27640116

[69] Fradi H, Dugelay J-L (2015) Spatial and temporal variations of feature tracks for crowd behavior analysis. Journal on Multimodal User Interfaces 10: 307–317.

[70] Wang B, Ye M, Li X, Zhao F, Ding J (2011) Abnormal crowd behavior detection using high-frequency and spatio-temporal features. Machine Vision and Applications 23: 501–511.

[71] Wang B, Ye M, Li X, Zhao F (2011) Abnormal crowd behavior detection using size-adapted spatio-temporal features. International Journal of Control, Automation and Systems 9: 905–912.

[72] Zeitz Kathryn M., Tan HM, Zeitz CJ (2009) Crowd Behavior at Mass Gatherings: A Literature Review. Prehospital and disaster medicine 24: 32–38. 19557955 10.1017/s1049023x00006518

[73] Ulicny B, Thalmann D (2002) Towards Interactive Real-Time Crowd Behavior Simulation. Computer Graphics Forum 21: 767–775.

