# Supplementary material for: Occurrence mechanism and coping paths of accidents of highly aggregated tourist crowds based on system dynamics
Source: PLoS One. 2019 Sep 17;14(9):e0222389. doi: 10.1371/journal.pone.0222389 (PMC6748560; doi:10.1371/journal.pone.0222389)
Supplement: S1 Ethics — (PDF) [file pone.0222389.s002.pdf]

PLOS ONE

Manuscript Title: A study on accidents of occurrence mechanism and coping paths of highly aggregated tourist crowds based on system dynamics

Manuscript ID: PONE-D-18-33069

Authors: Jie Yin, Xiang-min Zheng, Ruey-Chyn Tsaur

Ethics statement

On behalf of the authors of this paper and having obtained permission from all the authors, I declare that during collecting data through Baidu news and Sina Weibo, this study completely followed the user agreement and privacy policy of the website. The collection method also complied with the terms and conditions for the websites. Besides, all the personal information of tourists involved in the cases is protected.

I testify to the accuracy of the above on behalf of all the authors

Jie Yin Jie Yin

June 26, 2019 2019.06.26
